# Correspondence

**Published:** 1874-11

**Authors:** 


### Correspondence.

EDITORS CHICAGO MEDICAL JOURNAL:

Your correspondent who signs himself "A Regular," has struck a chord which shall vibrate through the land. It is the key-note of a coming reform. "You cannot eradicate quackery by such narrow-minded, unlogical restrictions as the code of ethics tries to force upon the regular physicians." So says "A Regular;" so say we; and so will say thousands of regular, true and good medical men all over this broad domain. From hundreds of hamlets, villages and towns, this word will come up to sustain "A Regular" in this his bold and manly assertion.

History tells every school-boy of Braddock's defeat, and how the brave young American soldier pointed out to him the true way of coping with the concealed and treacherous foe. The leaders and teachers of the "ethics" continue to illustrate the stupidity of Braddock, and they and we are constantly suffering the same defeats; worse than this, too, humanity bleeds, suffers and dies, because of this same stupidity. The many victims of disease are all about us; they plead for help; the cool, lying advertisement of the quack and charlatan promises them the wished-for boon. They accept of the proffered aid, take their nostrums and so-called "treatments," and—die, knowing of no "better way." And we, of a noble, humane profession, capable of being very angels of mercy to them, must stand with folded arms, silent and dignified, (?) bound by "the ethics." The dignity of "the profession" *must* be preserved, you know?!

COUNTRY PHYSICIAN—REGULAR.

